# Usefulness of the BIG Score in Predicting Massive Transfusion and In-Hospital Death in Adult Trauma Patients

**DOI:** 10.1155/2023/5162050

**Published:** 2023-10-17

**Authors:** Sejun Park, Il Jae Wang, Seok-Ran Yeom, Sung-Wook Park, Suck Ju Cho, Wook Tae Yang, Wonwoong Tae, Up Huh, Chanhee Song, Yeaeun Kim, Jong-Hwan Park, Youngmo Cho

**Affiliations:** ^1^Department of Emergency Medicine, School of Medicine, Pusan National University and Biomedical Research Institute, Pusan National University Hospital, Busan 49241, Republic of Korea; ^2^Department of Thoracic and Cardiovascular Surgery, Pusan National University School of Medicine and Biomedical Research Institute, Pusan National University Hospital, Busan 49241, Republic of Korea; ^3^Medical Research Institute, Pusan National University, Busan 49241, Republic of Korea; ^4^Department of Health Care Management, Catholic University of Pusan, Busan 46252, Republic of Korea; ^5^Health Convergence Medicine Laboratory, Biomedical Research Institute, Pusan National University Hospital, Busan 49241, Republic of Korea

## Abstract

The base deficit (B), international normalized ratio (I), and Glasgow coma scale (GCS) (BIG) score is useful in predicting mortality in pediatric trauma patients; however, studies on the use of BIG score in adult patients with trauma are sparse. In addition, studies on the correlation between the BIG score and massive transfusion (MT) have not yet been conducted. This study aimed to evaluate the predictive value of BIG score for mortality and the need for MT in adult trauma patients. This retrospective study used data collected between 2016 and 2020 at our hospital's trauma center and registry. The predictive value of BIG score was compared with that of the Injury Severity Score (ISS) and Revised Trauma Score (RTS). Logistic regression analysis was carried out to assess whether BIG score was an independent risk factor. Receiver operating characteristic (ROC) curve analysis was performed, and predictive values were evaluated by measuring the area under the ROC curve (AUROC). In total, 5,605 patients were included in this study. In logistic regression analysis, BIG score was independently associated with in-hospital mortality (odds ratio (OR): 1.1859; 95% confidence interval (CI): 1.1636–1.2086) and MT (OR: 1.0802; 95% CI: 1.0609–1.0999). The AUROCs of BIG score for in-hospital mortality and MT were 0.852 (0.842–0.861) and 0.848 (0.838–0.857), respectively. Contrastingly, the AUROCs of ISS and RTS for in-hospital mortality were 0.795 (0.784–0.805) and 0.859 (0.850–0.868), respectively. Moreover, AUROCs of ISS and RTS for MT were 0.812 (0.802–0.822) and 0.838 (0.828–0.848), respectively. The predictive value of BIG score for mortality and MT was significantly higher than that of the ISS. The BIG score also showed a better AUROC for predicting in-hospital mortality compared with RTS. In conclusion, the BIG score is a useful indicator for predicting mortality and the need for MT in adult trauma patients.

## 1. Introduction

Injuries represent a major public health concern, contributing significantly to morbidity and mortality worldwide [1]. The global burden of traumatic injuries is evident, with approximately 40 million people experiencing trauma annually in the United States alone and 6 million deaths reported worldwide [2]. In South Korea, the impact of traumatic injuries is no less alarming, as evidenced by the 2021 statistics results from the Korean Trauma Data Bank (KTDB), revealing 1,602 deaths among 8,906 severe trauma patients [3]. The severity of traumatic injuries necessitates the development of reliable tools and indicators to assess prognosis and guide treatment decisions for such patients [4].

Among the various scoring systems, the base deficit (BD), international normalized ratio (INR), and Glasgow coma scale (GCS) (BIG) score, which is derived from three key indicators known to influence the survival outcomes of trauma patients [5], have emerged as a promising tool because of its simplicity and clinical applicability. The BIG score has demonstrated utility in predicting outcomes among pediatric trauma patients. In addition, previous studies have demonstrated the encouraging results on the use of BIG score in predicting mortality in adult trauma patients when compared with those of other trauma assessment indicators [6, 7]. However, the literature on the use of BIG score for adult trauma patients remains relatively scarce, with only a limited number of studies addressing this topic. Moreover, studies assessing the correlation between the BIG score and massive transfusion (MT) have not yet been conducted.

Therefore, the primary objective of this study was to assess the predictive value of BIG score for mortality rates in adult trauma patients. The second objective of this study was to evaluate the predictive power of BIG score for MT in adult trauma patients. We hypothesized that the BIG score will serve as a valuable prognostic tool for predicting mortality and MT in adult trauma patients.

## 2. Materials and Methods

This was a retrospective single-center study conducted in a trauma center of a tertiary care university hospital. The hospital operates a separate emergency department within the trauma center, which is equivalent to a grade A trauma center representing the southeastern region of South Korea. Approximately, 1,000 severe trauma patients present at the trauma center annually. This study was approved by the Institutional Review Board (IRB) of our hospital (IRB 2306-003-127) and conducted in accordance with the relevant guidelines.

This study included patients who presented to the emergency department of the trauma center between 2016 and 2020. The exclusion criteria were as follows: (a) age <16 years, (b) cardiac arrest when admitted to the trauma center, and (c) missing values for the BIG score component, MT, and mortality.

Data were extracted from the KTDB and electronic medical records of the hospital. KTDB is a statistic data published annually by the National Emergency Medical Center in Korea that provides the basis for a trauma treatment system by collecting information on trauma patients from medical institutions selected as regional trauma centers and providing the information necessary for various studies [3]. The extracted data included age, sex, type of injury, Revised Trauma Score (RTS), MT, Injury Severity Score (ISS), in-hospital GCS score, lactic acid level, prothrombin time (PT)/INR, BD, pH, activated partial thromboplastin time (APTT), BIG score, and aspartate transaminase (AST) and alanine transaminase (ALT) levels.

The BIG score was calculated using the following formula:

(1) $$\begin{array}{c} \text{BIG score}=\left(\text{BD}\right)+\left(2.5\times \text{INR}\right)+\left(15-\text{GCS}\right). \end{array}$$

Categorical variables are reported as frequencies (percentages) and continuous variables as medians of the interquartile range. We performed receiver operating characteristic (ROC) analysis to identify the sensitivity, specificity, positive predictive value (PPV), negative predictive value (NPV), positive likelihood ratio (PLR), and negative likelihood ratio (NLR) of the BIG score. Predictive value was assessed using the area under the ROC (AUROC) curve. Logistic regression analysis was carried out to determine the independent effect of BIG score on mortality and MT. All tests were two-sided, and *P* values <0.05 were considered statistically significant. MedCalc version 22.007 (MedCalc Software, Ostend, Belgium) was used for the statistical analyses.

## 3. Results and Discussion

**Figure 1:**
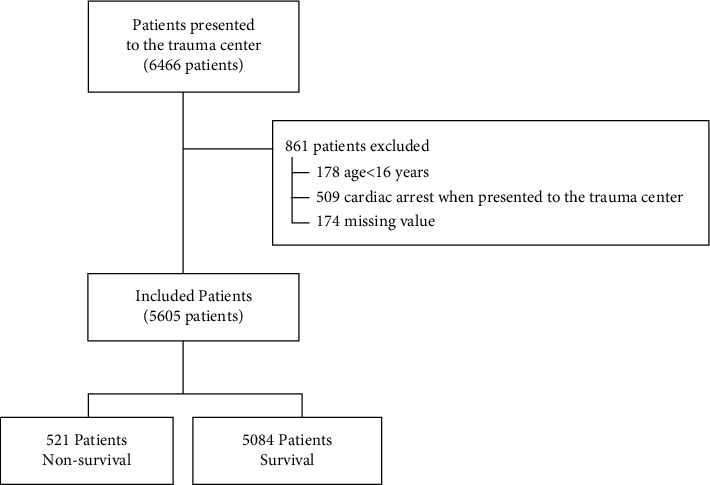
Study flow diagram.

During the study period, 6,466 patients visited the emergency department of the trauma center. Of these, 861 were excluded: age <16 years, *n* = 178; cardiac arrest at the time of emergency department visit, *n* = 509; and missing values, *n* = 174. Ultimately, 5,605 patients were included in this study (Figure 1) and 4,330 (77.25%) were male. The mean age of the patients was 58 years (range, 43–68). Among all patients, 521 (9.30%) died and 437 (7.8%) received MT.

We then compared the survival and nonsurvival groups. The nonsurvival group had a significantly lower GCS score (*P* < 0.001) and higher PT/INR and BD (*P* < 0.001) than the survival group. Furthermore, the nonsurvival group had a significantly lower pH (*P* < 0.001) and higher RTS, ISS, lactic acid level, APTT, and AST/ALT levels (*P* < 0.001) than the survival group. The BIG score and MT ratio were significantly higher in the nonsurvival group than in the survival group (*P* < 0.001). The data are presented in Table 1.

**Figure 2:**
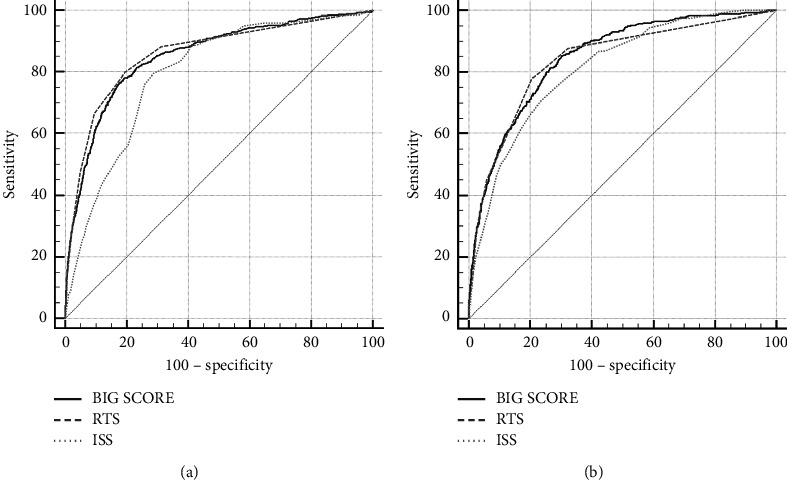
Receiver operating characteristic curve for in-hospital mortality (a) and massive transfusion (b).

ROC analysis was performed, and AUROC was measured to evaluate the predictive value (Figure 2). AUROCs of BIG score for in-hospital mortality and MT were 0.852 and 0.848, respectively (Table 2). AUROC of BIG score for in-hospital mortality was not significantly lower than that for the RTS and significantly higher than that for the ISS. In addition, AUROC of BIG score for MT was superior to that for both RTS and ISS. The sensitivity, specificity, PPV, NPV, PLR, and NLR of the BIG score, ISS, and RTS are shown in Table 3. To determine whether BIG score was an independent risk factor for in-hospital mortality and MT, logistic regression analysis was performed (Table 4). BIG score remained independently associated with in-hospital mortality (odds ratio (OR): 1.1859; 95% confidence interval (CI): 1.1636–1.2086; *P* < 0.001) and MT (odds ratio (OR): 1.0802; 95% confidence interval (CI): 1.0609–1.0999; *P* < 0.001) after adjustment for age, sex, and ISS.

## 4. Discussion

This study aimed to assess the ability of BIG score to predict in-hospital mortality and MT in adult trauma patients and compare its predictive value with that of ISS and RTS. The findings of this study demonstrated that the BIG score was an independent risk factor for in-hospital mortality and MT in adult trauma patients. Moreover, the prognostic value of BIG score for mortality and MT was significantly higher than that of the ISS, and the BIG score also showed a better AUROC for predicting in-hospital mortality than the RTS.

Considered the most well-known pediatric trauma scoring system, the Pediatric Trauma Score consists of six items: weight, systolic blood pressure, external wounds, fractures, airway, and level of consciousness. Another widely used pediatric scoring system, the Pediatric Risk Index, requires 12 parameters [5]. These scoring tools used for children were greatly complicated, with difficulty in identifying results rapidly; thus, a simpler scoring tool was required to predict outcomes in pediatric trauma patients. The BIG score, introduced in 2010 in war and civilian sectors, eliminates anatomical and hemodynamic parameters. Instead, it utilizes parameters such as the GCS, which is a simple clinical evaluation, and laboratory parameters BE and PT/INR, which can be easily obtained in the emergency department [5]. Unlike other scoring tools, such as the ISS, the BIG score does not require variables that are not readily available during the acute phase of injury management.

The most common cause of death in adult trauma patients is traumatic brain injury, followed by hemorrhage [8, 9]. GCS is an effective tool for predicting mortality as it represents the severity of traumatic brain injury. Several studies have highlighted the value of GCS as a component of BIG score when evaluating the correlation between the severity of traumatic brain injury and patient outcomes [10, 11]. GCS is still widely used in trauma care settings and is an important factor in predicting mortality and guiding treatment decisions. Traumatic hemorrhage, the second leading cause of traumatic mortality, causes acidosis and coagulopathy. The BIG score uses BD to reflect shock and acidosis. BD is a valuable predictor of shock, abdominal injury, fluid demand, resuscitation effectiveness, and post-traumatic mortality in trauma patients [12–17]. In particular, BD, as an independent variable in adult trauma patients, is an effective factor in predicting trauma patient mortality [12, 18–20]. The INR component of the BIG score represents the patient's coagulation activity. In trauma patients, coagulopathy can occur owing to an increase in the INR, leading to impaired blood clotting and increased bleeding risk. The mechanisms underlying coagulopathy can be complex and multifactorial, involving various physiological responses to injury and activation of the coagulation system. Several factors contribute to the increase in INR in trauma patients [21]. Accordingly, coagulopathy is an important factor to predict death in trauma patients [14, 22]. Therefore, the BIG score has a strong predictive power for mortality in trauma patients because it includes parameters that reflect the two major causes of acute death from trauma (brain damage and bleeding).

Two previous studies have validated the predictive ability of the BIG score for assessing the mortality of adult patients with trauma. In the first study, MacLeod et al. compared the mortality prediction of BIG score, Trauma Score and ISS (TRISS), and PS09 in 4,949 patients in a private trauma center and 7,257 patients in a military trauma center, with AUROCs of 0.892 (0.879–0.906), 0.922 (0.913–0.932), and 0.925 (0.915–0.934), respectively [22]. In the second study by Höke et al., the predictive abilities for mortality of the BIG score, TRISS, new ISS, ISS, RTS, and GCS were compared. The resulting AUROC values were 0.87 (0.84–0.90), 0.94 (0.91–0.98), 0.88 (0.84–0.91), 0.85 (0.81–0.88), 0.81 (0.77–0.85), and 0.79 (0.75–0.83), respectively [7]. AUROC of BIG score (0.852 (0.842–0.861)) in the present study was similar to that of the previous studies.

According to our results, the BIG score showed good outcomes in predicting mortality and the need for MT. To the best of our knowledge, this is the first study to assess the role of BIG score as a predictor for MT in adult trauma patients. The early and accurate prediction of MT is crucial in trauma patients because of several reasons. First, early intervention allows early preparation and rapid initiation of transfusion therapy. Moreover, large amounts of blood products and resources may be required, and by predicting these needs, healthcare providers can allocate and efficiently supply these resources. Second, patient management through risk stratification and appropriate treatment planning can be established. Finally, patients planned for particularly large blood transfusions may require a more aggressive surgical approach or immediate access to specialized facilities. Early identification of these patients aids in surgical planning and decision-making. Hence, in time-sensitive situations, predicting the need for massive blood transfusion in trauma patients can enable faster decision-making and intervention, potentially saving lives in critical situations.

This study had several limitations. First, owing to its retrospective nature and the use of registry data, we were unable to identify any potential medical errors that could have influenced the treatment outcomes. Second, this study was conducted in a single center, which might limit the generalizability of the findings to patients in other emergency departments. Third, scores reflecting the results of Focused Assessment with Sonography in Trauma, such as the Assessment of Blood Consumption score, were not used in this study because no ultrasound results were available in the trauma center's database.

## 5. Conclusions

The BIG score is a valuable indicator for predicting both mortality and the need for MT in adult trauma patients. The predictive value of the BIG score surpassed that of the ISS and was comparable to that of the RTS, making it a powerful tool for adult trauma assessment.

## Figures and Tables

**Table 1:** Characteristics of included patients.

Variables	Total (*n* = 5605)	Nonsurvival (*n* = 521)	Survival (*n* = 5084)	*P* value
Age (y), median (IQR)	58 (43, 68)	65 (55, 77)	57 (42, 68)	<0.0001
Sex, *n* (%)				<0.0001
Male	4330 (77.25)	390 (74.86)	3940 (77.5)	
Female	1275 (22.75)	131 (25.14)	1144 (22.5)	
Type of injury, *n* (%)				<0.0001
Traffic accident	2835 (50.58)	275 (52.78)	2560 (50.36)	
Fall from height	1527 (27.24)	149 (28.6)	1378 (27.1)	
Ground level fall	301 (5.37)	27 (5.18)	274 (5.39)	
Object blunt	427 (7.62)	23 (4.42)	404 (7.95)	
Penetrating	363 (6.48)	7 (1.34)	356 (7)	
Others	152 (2.71)	40 (7.68)	112 (2.2)	
Red blood cells—blood transfused within 4 hours of hospital visit, median (IQR)	0 (0, 2)	4 (1, 9)	0 (0, 1)	<0.0001
Red blood cells—blood transfused within 24 hours of hospital visit, median (IQR)	0 (0, 1)	1 (0, 5)	0 (0, 2)	<0.0001
Massive blood transfusion, *n* (%)				<0.0001
No	5168 (92.2)	325 (62.38)	4843 (95.26)	
Yes	437 (7.8)	196 (37.62)	241 (4.74)	
ISS, median (IQR)	17 (10, 26)	29 (25, 38)	17 (10, 25)	<0.0001
Hospital GCS, median (IQR)	15 (12, 15)	5 (3, 9)	15 (14, 15)	<0.0001
Lactic acid median (IQR)	2.5 (1.6, 4.1)	4.4 (2.6, 8.8)	2.4 (1.5, 3.8)	<0.0001
PT INR, median (IQR)	1.06 (1, 1.16)	1.26 (1.11, 1.63)	1.05 (0.99, 1.13)	<0.0001
Base deficit, median (IQR)	0.5 (−1.8, 3.9)	4.4 (0.5, 10.8)	0.4 (−2.1, 3.4)	<0.0001
pH, median (IQR)	7.4 (7.36, 7.44)	7.35 (7.19, 7.42)	7.41 (7.36, 7.44)	<0.0001
APTT_TIME, median (IQR)	26.7 (24.1, 30.6)	34.3 (27.9, 45.6)	26.4 (23.9, 30)	<0.0001
BIG score	4.48 (1.23, 10.45)	16.83 (11.45, 24.15)	3.83 (0.95, 8.65)	<0.0001
AST, median (IQR)	43 (28, 96)	66.5 (39, 169.75)	42 (27, 89)	<0.0001
ALT, median (IQR)	31 (20, 59)	38 (23, 79)	30 (20, 58)	<0.0001

IQR, interquartile range; ISS, Injury Severity Score; GCS, Glasgow coma scale; PT_INR, prothrombin international normalized ratio; pH, percentage of hydrogen ion; APTT, activated partial thromboplastin time; BIG, base deficit, international normalized ratio, and Glasgow coma scale; AST, aspartate transaminase; ALT, alanine transaminase.

**Table 2:** AUROC for the BIG score, ISS, and RTS regarding in-hospital mortality and MT.

	AUROC (95% CI)	*P* value	*P* value (compared to BIG score)
In-hospital mortality			
BIG score	0.852 (0.842–0.861)	*P* < 0.0001	—
ISS	0.795 (0.784–0.805)	*P* < 0.0001	*P* < 0.0001
RTS	0.859 (0.850–0.868)	*P* < 0.0001	*P* = 0.3085
Massive blood transfusion			
BIG score	0.848 (0.838–0.857)	*P* < 0.0001	—
ISS	0.812 (0.802–0.822)	*P* < 0.0001	*P* = 0.0025
RTS	0.838 (0.828–0.848)	*P* < 0.0001	*P* = 0.2505

BIG: base deficit, international normalized ratio, and Glasgow coma scale; ISS: Injury Severity Score; RTS: Revised Trauma Score; AUROC: area under the receiver operating characteristics curve.

**Table 3:** Sensitivity, specificity, PPV, NPV, PLR, NLR, and AUROC for BIG score, ISS, and RTS.

	BIG score	ISS	RTS	*P* value
In-hospital mortality				<0.0001
Sensitivity (95% CI)	76.39 (72.5–80.0)	79.46 (75.7–82.9)	79.85 (76.1–83.2)	
Specificity (95% CI)	82.51 (81.3–83.4)	71.22 (70.0–72.5)	80.52 (79.4–81.6)	
Positive predictive value, %	30.7 (29.1–32.4)	22.1 (21.0–23.1)	29.6 (28.1–31.1)	
Negative predictive value, %	97.1 (96.7–97.5)	97.1 (96.6–97.6)	97.5 (97.0–97.9)	
Positive likelihood ratio	4.33 (4.01–4.67)	2.76 (2.60–2.94)	4.10 (3.82–4.40)	
Negative likelihood ratio	0.29 (0.25–0.33)	0.29 (0.24–0.34)	0.25 (0.21–0.30)	
AUROC	0.852 (0.842–0.861)	0.795 (0.784–0.805)	0.859 (0.850–0.868)	
Massive blood transfusion				<0.0001
Sensitivity (95% CI)	85.35 (81.7–88.5)	70.25 (65.7–74.5)	78.03 (73.9–81.8)	
Specificity (95% CI)	69.60 (67.9–70.5)	77.03 (75.9–78.2)	79.39 (78.3–80.5)	
Positive predictive value, %	19.0 (18.1–19.9)	20.5 (19.3–21.9)	24.3 (22.9–25.6)	
Negative predictive value, %	98.2 (97.8–98.6)	96.8 (96.4–97.3)	97.7 (97.3–98.1)	
Positive likelihood ratio	2.77 (2.62–2.93)	3.06 (2.83–3.31)	3.79 (3.52–4.07)	
Negative likelihood ratio	0.21 (0.17–0.27)	0.39 (0.33–0.45)	0.28 (0.23–0.33)	
AUROC	0.848 (0.838–0.857)	0.812 (0.802–0.822)	0.838 (0.828 to 0.848)	

PPV: positive predictive value; NPV: negative predictive value; PLR: positive likelihood ratio; NLR: negative likelihood ratio; BIG: base deficit, international normalized ratio, and Glasgow coma scale; ISS: Injury Severity Score; RTS: Revised Trauma Score; AUROC: area under the receiver operating characteristics curve.

**Table 4:** Multiple logistic regression analysis for in-hospital mortality and MT.

Variables	In-hospital mortality		Massive blood transfusion	
	OR (95% CI)	*P* value	OR (95% CI)	*P* value
Age (years)	1.0423 (1.0352–1.0495)	<0.0001	1.0073 (1.0010–1.0136)	0.0239
Sex (male)	1.0798 (0.8333–1.3993)	0.5614	0.7543 (0.5816–0.9781)	0.0334
ISS	1.0503 (1.0404–1.0604)	<0.0001	1.0655 (1.0553–1.0758)	<0.0001
Big score	1.1859 (1.1636–1.2086)	<0.0001	1.0802 (1.0609–1.0999)	<0.0001

MT: massive transfusion; OR: odds ratio; CI: confidence interval; ISS: Injury Severity Score.

## Data Availability

The datasets used and/or analyzed during the current study are available from the corresponding author on reasonable request.
